# Species of the Sections *Hedysarum* and *Multicaulia* of the Genus *Hedysarum* (Fabaceae): Taxonomy, Distribution, Chromosomes, Genomes, and Phylogeny

**DOI:** 10.3390/ijms25158489

**Published:** 2024-08-03

**Authors:** Olga Yu. Yurkevich, Tatiana E. Samatadze, Svyatoslav A. Zoshchuk, Alexandra V. Amosova, Olga V. Muravenko

**Affiliations:** Engelhardt Institute of Molecular Biology, Russian Academy of Sciences, 32 Vavilov St, 119991 Moscow, Russia

**Keywords:** *Hedysarum*, *Multicaulia*, nuclear and plastid DNA sequences, genome, chromosome, repeatome, molecular markers, rDNAs, satDNAs, NGS, FISH

## Abstract

The genus *Hedysarum* L. (Fabaceae) includes about 200 species of annual and perennial herbs distributed in Asia, Europe, North Africa, and North America. Many species of this genus are valuable medicinal, melliferous, and forage resources. In this review, we consider the taxonomic history of the genus *Hedysarum*, the chromosomal organization of the species from the sections *Hedysarum* and *Multicaulia*, as well as phylogenetic relationships between these sections. According to morphological, genetic, and phylogenetic data, the genus *Hedysarum* is divided into three main sections: *Hedysarum* (= syn. *Gamotion*), *Multicaulia*, and *Stracheya*. In species of this genus, two basic chromosome numbers, x = 7 (section *Hedysarum*) and x = 8 (sections *Multicaulia* and *Stracheya*), were determined. The systematic positions of some species within the sections are still uncertain due to their morphological similarities. The patterns of distribution of molecular chromosomal markers (45S rDNA, 5S rDNA, and different satellite DNAs) in karyotypes of various *Hedysarum* species made it possible to determine their ploidy status and also specify genomic relationships within the sections *Hedysarum* and *Multicaulia.* Recent molecular phylogenetic studies clarified significantly the taxonomy and evolutionary development of the genus *Hedysarum.*

## 1. Introduction

The genus *Hedysarum* L. (Fabaceae) involves about 200 species of annual and perennial herbs distributed in Asia, Europe, North Africa, and North America [1,2]. Species of this genus are well known as medicinal, melliferous, and forage resources. Many plants of the genus *Hedysarum* contain valuable biologically active compounds that are important for obtaining new drugs with therapeutic properties against viral, oncological, neurological, and cardiovascular diseases [3,4,5,6,7,8]. Several *Hedysarum* species (*H. flavescens* Rgl. et Schmalh., *H. gmelinii* Ledeb., and others) are characterized by high biomass productivity in summer, and they are widely used as forage plants [1,9,10]. Some species, including *H. grandiflorum* Pall., *H. ucrainicum* Kaschm., and *H. coronarium* L., bloom profusely, and they are common honey plants in Eurasia [11,12].

Currently, the genus *Hedysarum* consists of three sections: *Hedysarum* (= syn. *Gamotion*), *Multicaulia*, and *Stracheya* [13,14,15]. However, the systematic positions of some species within the sections of this genus are still uncertain due to their high morphological similarities. Considerable variability in morphological features observed among the related species, especially in the areas where their ranges overlap, often prevents their accurate identification. In particular, *H. arcticum* B. Fedtsch. is described either as an independent plant species [1,16] or as a subspecies of *H. hedysaroides* (L.) Schinz et Thell. [17,18]. The species *H. setigerum* Turcz. ex Fisch. et Meyer and *H. gmelinii* Ledeb. are rather similar in morphological characters, and taxonomists identify *H. setigerum* either as a separate species [1] or as a subspecies of *H. gmelinii* [16,19].

The analysis of genetic diversity within the genus *Hedysarum* by means of molecular AFLP and ISSR markers revealed a high level of intraspecific polymorphisms [20,21,22,23]. However, genetic polymorphism studies in populations of East European (*H. grandiflorum*, *H. biebersteinii* Zertova, and *H. argyrophyllum* Ledeb.) and South Siberian (*H. setigerum* and *H. gmelinii*) species of the section (sect.) *Multicaulia*, performed based on molecular genetic (ISSR) markers, could not distinguish species with overlapping ranges [24,25]. Using protein-based molecular markers, reliable differentiation was demonstrated only between two closely related species (*H. neglectum* Ledeb. and *H. theinum* Krasnob.) from the sect. *Hedysarum* [26,27].

Interspecific karyotypic differences within the sections were also not found by monochrome staining of chromosomes. In karyotypes of *Hedysarum* species, two basic chromosome numbers x = 7 (section *Hedysarum*) and x = 8 (sections *Multicaulia* and *Stracheya*) were determined [2,13,28,29]. Although other basic numbers were not revealed within the genus, the peculiarities of inter- and intraspecific chromosomal variability, ploidy status, and also relationships between genomes of the species from different sections, still remain insufficiently studied.

The phylogeny of the genus *Hedysarum* is also controversial. In this genus, three well-supported clades were recognized according to the molecular phylogenetic studies using nuclear (ITS) and plastid (psbA-trnH, trnL—trnF, and matK) DNA sequences [13,14,15]. Based on the results of the phylogenetic studies, both paraphyletic and monophyletic origins of the genus were assumed [13,14,15,30]. For more substantiated conclusions regarding the evolutionary development of the genus *Hedysarum*, further investigation of the species genomes using various modern approaches is required.

In this review, we consider the taxonomic history of the genus *Hedysarum*, the chromosomal organization of the species from the sections *Hedysarum* and *Multicaulia*, as well as phylogenetic relationships between these sections. We review recent karyogenomic and molecular genetic studies of species of the genus *Hedysarum*, which make it possible to identify individual chromosomes, analyze the nature of chromosome number variability in *Hedysarum* species, and the pathways of chromosomal reorganization that occurred in their genomes during speciation, as well as the relationships within and between the sections of this genus.

## 2. Comparative Analysis of the Species from the Sections *Hedysarum* and *Multicaulia*

### 2.1. Taxonomy and Distribution in Eurasia

The history of taxonomic revisions of the genus *Hedysarum* is quite complicated. The first classification of *Hedysarum* was made by Linnaeus (1753) [31]. He subdivided the genus *Hedysarum* into four sections according to plant leaf shapes. Later, Candolle (1825) and Boissier (1872) classified *Hedysarum* species based on the presence or absence of bristles and setae on the joints of the pod as well as on the habit of the species [32,33]. Fedchenko (1902) was the first botanist who tried to summarize the available data on the morphology and taxonomy of *Hedysarum* and attempted to revise the species boundaries [34]. He produced a new classification based on the life forms of plants, and also on the characteristics of their vegetative and generative organs [1,34]. Fedchenko subdivided the genus *Hedysarum* into seven sections: the *H*. sect. *Crinifera* (Boiss.) B. Fedtsch., *H*. sect. *Fruticosa* B. Fedtsch., *H.* sect. *Membranacea*, *H.* sect. *Multicaulia* (Boiss.) B. Fedtsch., *H.* sect. *Obscura* B. Fedtsch., *H.* sect. *Spinosissima* B. Fedtsch., and *H.* sect. *Subacaulia* (Boiss.) B. Fedtsch [1]. According to Fedchenko’s classification, *Subacaulia* and *Multicaulia* were classified into different sections, which is still relevant today [1,9].

In a recent revision of the taxonomy of the genus *Hedysarum*, several sections, proposed in Fedchenko’s classification, were combined or transferred to other genera, and the genus *Hedysarum* was divided into four sections: the *H.* sect. *Hedysarum*, *H.* sect. *Membranacea*, *H.* sect. *Multicaulia*, and *H.* sect. *Stracheya*. In particular, the sections *Multicaulia*, *Crinifera*, and *Subacaulia* were combined into one section, *Multicaulia*, *H.* sect. *Obscura* was renamed as *H.* sect. *Hedysarum*, and the monotypic genus *Stracheya* Benth. was moved to *Hedysarum* as sect. *Stracheya* [2]. Current classifications, which are based on molecular data, divide the genus *Hedysarum* into three sections: *Hedysarum*, *Multicaulia*, and *Stracheya*, and the sect. *Multicaulia* includes the subsections *Multicaulia* and *Crinifera* [13,14,15,35].

The sect. *Hedysarum* is one of the widest-ranging sections within the genus *Hedysarum*. The species belonging to this section are distributed in temperate and boreal regions of the Northern Hemisphere. They usually grow in alpine and arctic meadows, stone grasslands, deserts, and seashores [1,2]. Among all studied species from the sect. *Hedysarum*, *H. alpinum* L. has the widest distribution range which covers Europe, Siberia, the Far East, Northern Mongolia, China, and the Korean Peninsula [1,16] and overlaps with the habitats of *H. theinum* and *H. neglectum*. At the same time, these species occupy different ecological niches [9,16]. The species *H. flavescens* Rgl. et Schmalh. occupies a narrow ecological niche growing on the limited areas within the mountain ranges of the Western Tian Shan and Pamir-Alay Mountains [10]. *H. flavescens* and other yellow-flowering species of the sect. *Hedysarum* is considered to be a primary mesophilic group distributed in the highlands of the Pamir-Alay and Tien Shan Mountains [36].

Most species from the sect. *Multicaulia* are distributed in Southern Siberia and Central Asia [1,2]. For example, *H. grandiflorum* Pall., *H. razoumovianum* Fisch. et Helm ex DC., and *H. gmelinii* grow in the Eastern European region. *H. zundukii* Peschkova, *H. dahuricum* (Turcz.) B. Fedtsch., *H. setigerum*, and *H. gmelinii* are distributed in the Southern Siberian region. This means that *H. gmelinii* can grow in both regions and its range overlaps with all studied species [1,9,16]. *H. grandiflorum*, *H. razoumowianum*, and *H. zundukii* are rare and endangered taxa [37,38]. At the same time, *H. grandiflorum* is a wide-ranging species, and both *H. razoumowianum* and *H. zundukii* occupy narrow areas [1,38]. The ranges of *H. setigerum* and *H. gmelinii* are partially overlapped, and the morphological similarities between these closely related species make their taxonomies difficult [1,16,19].

**Figure 1:**
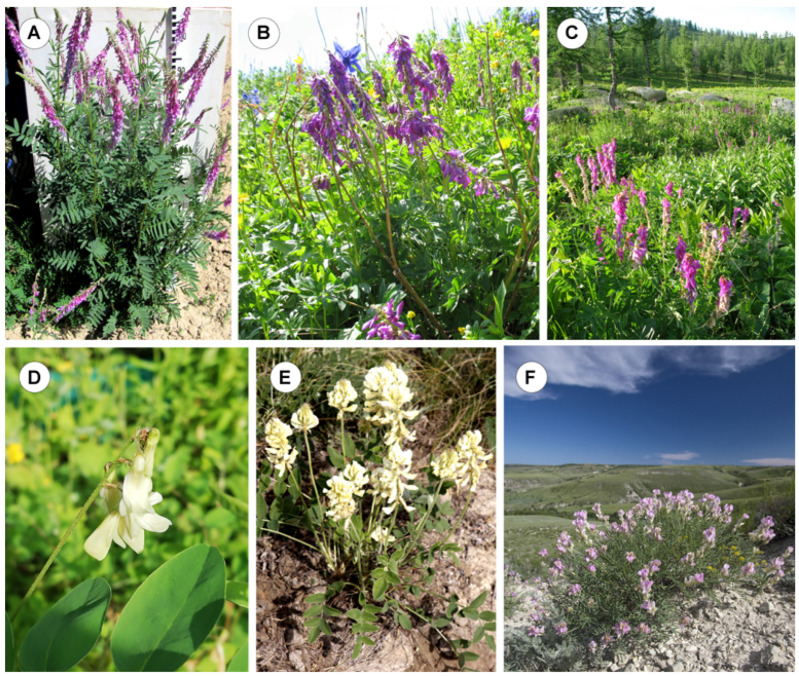
Plants growing on the trial plot (AIMAP, Moscow) and wild populations of some *Hedysarum* species. *H. alpinum* (the trial plot of AIMAP, Moscow, Russia) (**A**), *H. neglectum* (the Altai region, Russia) (**B**), *H. theinum* (Kazakhstan) (**C**), *H. flavescens* (the trial plot of AIMAP, Moscow, Russia) (**D**), *H. grandiflorum* (Volgograd region, Russia) (**E**), and *H. razoumovianum* (Volgograd region, Russia) (**F**). The figure is adapted from “Molecular Cytogenetics of Eurasian Species of the Genus *Hedysarum* L. (Fabaceae)” by Yurkevich et al., 2021, *Plants*, 10, 89 [29] and “Integration of Genomic and Cytogenetic Data on Tandem DNAs for Analyzing the Genome Diversity Within the Genus *Hedysarum* L. (Fabaceae)” by Yurkevich et al., 2022, *Frontiers in Plant Science*, *13*, 865958 [39].

In Figure 1, several *Hedysarum* species growing on the trial plot or in their natural habit are presented.

### 2.2. Medicine Value of Hedysarum Species

The biological compounds revealed in *Hedysarum* medicinal species have immunomodulatory, antioxidant, antitumor, and antidiabetic effects [3,5,7,40,41]. A total of 155 biologically active substances including various amino acids carbohydrates, alkaloids, sterols, flavonoids, isoflavones, xanthones, tannins, and essential oils, were identified in their leaves and roots [4,5,6,7,8,42]. The species from the sections *Hedysarum* and *Multicaulia* are rich in xanthone magniferine and oligomeric catechins, which makes them valuable sources for the production of multifunctional biologically active substances and contributes to the development of new effective herbal medicines with antiviral and antibacterial properties [5,6,7,8,43,44,45]. *Hedysarum* species are popular in traditional medicine. In particular, leaves and stems of *H. alpinum* contain xanthon mangiferin, and the antiviral drug Alpizarin (Pharmacy center VILAR, Russia) is currently produced from this plant. The antiviral activity of the *H. alpinum* extract against influenza virus A/Aichi/2/68 (H3N2) (human) and A/chicken/Kurgan/05/2005 (H5N1) (birds) was demonstrated in transplanted culture of MDCK cells [46]. The analysis of biochemical parameters of rat blood in response to myocardial damage and also a model study of myocardial stabilization under stress showed that the herb extract of *H. alpinum* simultaneously possessed cardioprotective and antioxidant activity due to the presence in its composition of oxybenzoic and oxycoric acids [47]. Biologically active substances contained in extracts of *H. neglectum* roots showed antimicrobial and antioxidant activity, and plant root culture extracts have antagonistic activity against pathogenic and conditionally pathogenic strains [48]. Several species from the sect. *Multicaulia* (*H. gmelinii*, *H. grandiflorum*, and *H. setigerum*) are also promising sources for obtaining antiviral and antibacterial plant substances, such as xanthon mangiferin [43,45]. Some chalcones isolated from the roots of *H. gmelinii*, showed moderate antiproliferative activity against selective human cancer cell lines (HepG2, A549, Du145, BGC823, and HCT116) and also demonstrated in vitro anti-inflammatory activity [49]. Ethyl acetate extract of *H. candidissimum* Freyn showed a strong cytotoxic effect on HT-29 and MDA-MB-453 cancer cell lines [50]. At the same time, the natural resources of *Hedysarum* species are insufficient for ever-growing needs, and *H. alpinum*, *H. theinum*, *H. gmelinii*, and *H. grandiflorum* are already being cultivated and/or introduced into the cell culture using biotechnological techniques [51,52,53,54].

Roots of *H. polybotrys* Hand.-Mazz. are widely used in traditional Chinese medicine (TCM) to improve health conditions and treat various diseases. *Calycosin* (CA), a bioactive phytoestrogen isoflavone derived from *Hedysarum Radix* (the dried root of *Hedysarum polybotrys),* has potential effects as an anti-metastatic agent in various tumors, promoting apoptosis in cancer cells and exhibiting low toxicity to normal cells [55]. *Hedysarum* polysaccharides (HPS) are the most important natural active ingredients of *Hedysarum*, which have many pharmacological effects [8]. Additionally, HPS is the principal active fraction responsible for the antidiabetic properties. It is believed that HPS3 may partly ameliorate hyperglycemia and hyperlipidemia associated with type 2 diabetes through increased insulin secretion, inhibition of lipid peroxidation, promotion of sensitivity to insulin, suppression of gluconeogenesis, and reduction in the biosynthesis of fatty acids, cholesterol, and cell cytokines related to insulin resistance [3]. Moreover, anti-DPN (diabetic peripheral neuropathy) effects of HPS in genetically obese (ob/ob) mice were revealed [56]. HPS-MC (80%) had a prominent potential immune response under cyclophosphamide (CP)-induced immunosuppressive conditions in mice models [57]. HPS-50 showed a strong hepatoprotective effect after lipopolysaccharide (LPS)/D-galactosamine (D-GalN)-induced acute liver injury (ALI) in mice [58]. Currently, the main challenge in HPS research is to identify its specific components and their possible mechanisms of action. HPS exerts various pharmacological effects; however, the precise control of its dosage needs further study [8].

### 2.3. Karyological Studies of the Species from the Sections Hedysarum and Multicaulia

Karyological studies of species of the genus *Hedysarum* were mostly performed based on monochrome chromosome staining, and, for some endemic species, chromosome numbers are still unknown. The species from the genus *Hedysarum* are known to have small chromosome sizes (2–5 µm) and similar morphology [29,39,59,60]. The diploid species from the sect. *Hedysarum* have 2n = 14 chromosomes, while the species from the sect. *Multicaulia* have 2n = 16 chromosomes [2,13,28,29,60,61]. In karyotypes of some species of the genus *Hedysarum*, different chromosome numbers were detected. For example, 2n = 14, 28 for *H. arcticum* (sect. *Hedysarum*) [62] and 2n = 16, 32 for *H. dasycarpum* Turcz. (sect. *Multicaulia*) [63,64] were determined. In karyotypes of *H. setigerum* and *H. gmelinii*, 2n = 14, 28, 32, and 56 were revealed [16,65]. Recently, using a molecular cytogenetic approach, three levels of ploidy have been demonstrated in various *H. gmelinii* specimens (2n = 2x = 16, 2n = 4x = 32, and 2n = 6x = 48) [39].

In some karyotypes of tetraploid *H. gmelinii* and *H. setigerum*, and also in diploid *H. zundukii* and *H. sangilense* Krasnoborov et Timokhina, supernumerary small chromosomes (B chromosomes) were detected [39,63]. At the same time, the presence of additional chromosomes complicated the determination of a chromosome number in *Hedysarum* karyotypes, and simple monochrome staining did not allow for chromosome identification. Further studies of chromosome C-banding patterns performed in karyotypes of *H. coronarium* L. and *H. pallidum* Desf., revealed three types of bands (terminal, intercalary, and pericentromeric) [66,67]. In different populations of *H. perrauderianum* Coss. (2n = 32 + B), only small intercalary and terminal C-bands were detected, and, based on chromosome morphology and C-banding patterns, all chromosomes were identified [59]. In this species, CMA differential staining detected positive bands in centromeric regions of some chromosomes, and also in the NORs of the satellite chromosomes [59]. In karyotypes of Algerian species of *Hedysarum* (*H. carnosum* Desf., *H. spinosissimum* L., *H. pallidum* Desf., *H. perrauderianum*, and *H. naudinianum* Coss.), FISH-based chromosome mapping of 45S and 5S rDNA sequences and subsequent chromosome identification were carried out [59,60]. It was shown that 45S rDNA clusters were localized in one or two chromosome pairs, and also different numbers and positions of 5S rDNA clusters (in one or two pairs) were detected in karyotypes of *H. carnosum*, *H. spinosissimum*, *H. pallidum*, *H. perrauderianum*, and *H. naudinianum* [59,60].

**Figure 2:**
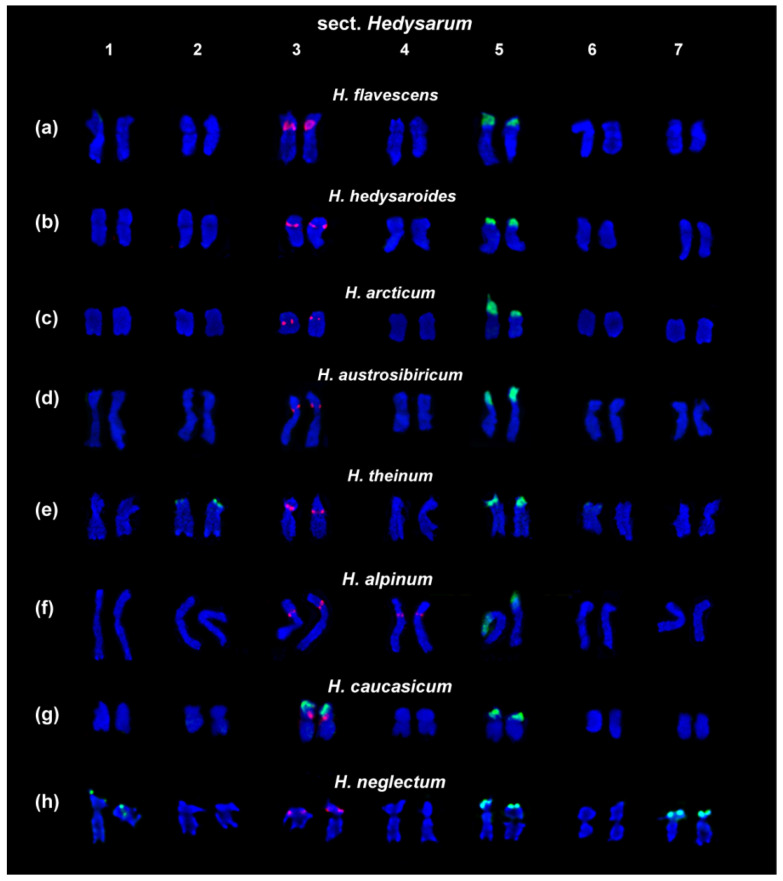
Karyotypes of the studied accessions of species from the sect. *Hedysarum*. Karyograms of *H. flavescens* (**a**), *H. hedysaroides* (**b**), *H. arcticum* (**c**), *H. austrosibiricum* (**d**), *H. theinum* (**e**), *H. alpinum* (**f**), *H. caucasicum* (**g**), and *H. neglectum* (**h**) after FISH with 45S rDNA (green) and 5S rDNA (red). Chromosome DAPI-staining—blue. The figure is adapted from “Molecular Cytogenetics of Eurasian Species of the Genus *Hedysarum* L. (Fabaceae)” by Yurkevich et al., 2021, *Plants*, 10, 89 [29].

Recent studies of karyotypes of the Eurasian species from the sect. *Hedysarum* (n = 7) and *Multicaulia* (n = 8) revealed inter- and intraspecific variability in patterns of chromosome localization of 45S and 5S rDNA clusters [29,39]. FISH-based chromosome mapping of 45S and 5S rDNA sequences in karyotypes of the diploid species with 2n = 14 (*H. alpinum*, *H. hedysaroides*, *H. arcticum*, *H. austrosibiricum*, *H. theinum*, and *H. flavescens*) detected one chromosome pair (5) bearing 45S rDNA clusters and one chromosome pair (3) with 5S rDNA clusters (Figure 2a–h). Moreover, in *H. theinum*, polymorphic minor 45S rDNA loci on chromosome pair 2 were observed (Figure 2e). Therefore, rDNA clusters could be effective molecular chromosomal markers that facilitate precise identification of morphologically similar species from the sect. *Hedysarum.*

In karyotypes of *H. neglectum*, *H. caucasicum*, and *H. alpinum*, species-specific unique marker variants of chromosome localization of 45S and 5S rDNA clusters were revealed. In *H. neglectum* the second major cluster of 45S rDNA was detected on satellite chromosome pair 7, in the karyotype of *H. caucasicum*, both 5S and 45S rDNA clusters were localized in the second satellite chromosome pair (3), and, in *H. alpinum*, the second chromosome pair with 5S rDNA (4) was found. These species-specific chromosome markers can be used for taxonomic research within the sect. *Hedysarum* [29,39].

**Figure 3:**
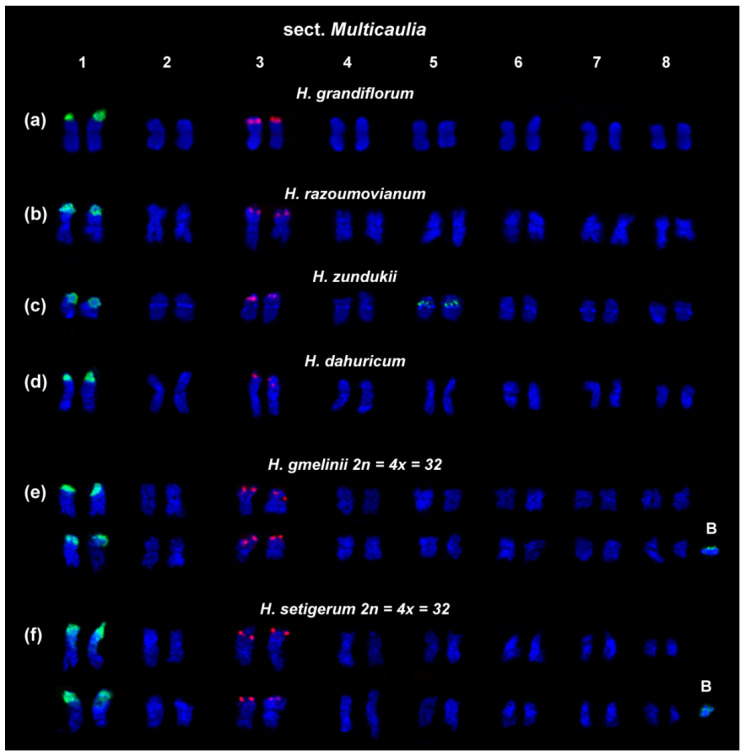
Karyotypes of the studied species accessions from the sect. *Multicaulia*. Karyograms of the studied accessions of diploid *H. grandiflorum* (**a**), *H. razoumovianum* (**b**), *H. zundukii* (**c**), *H. dahuricum* (**d**), and also tetraploid *H. gmelinii* (**e**), and *H. setigerum* (**f**) after FISH with 45S (green) and 5S (red) rDNA. B—B chromosomes. DAPI chromosome staining—blue. The figure is adapted from “Integration of Genomic and Cytogenetic Data on Tandem DNAs for Analyzing the Genome Diversity Within the Genus *Hedysarum* L. (Fabaceae)” by Yurkevich et al., 2022, *Frontiers in Plant Science*, *13*, 865958 [39].

The patterns of chromosomal distribution of major 45S and 5S rDNA clusters observed in the species from the sect. *Multicaulia* differed from those revealed in species from the sect. *Hedysarum* [29,39]. In karyotypes of the diploid species from the sect. *Multicaulia* (*H. grandiflorum*, *H. zundukii*, *H. dahuricum*, and *H. razoumovianum*), a major cluster of 45S rDNA was localized in the distal part of the short arms of the longest chromosome pair 1; and clusters of 5S rDNA were observed in the distal part of the short arms of chromosome pair 3 (Figure 3a–d). The tetraploid karyotypes of closely related *H. gmelinii* and *H. setigerum* were represented by two similar sets of chromosomes with the same chromosome distribution patterns of 45S and 5S rDNA indicating their tetraploid origin (2n = 4x = 32). In karyotypes of *H. zundukii*, minor species-specific loci of 45S rDNA were detected in the pericentromeric region of chromosome pair 5 [39].

In karyotypes of tetraploid *H. gmelinii* and *H. setigerum*, and also diploid *H. zundukii*, 1–3 supernumerary chromosomes (B chromosomes) were revealed. They were about 1 μm in length. DAPI-positive bands and also small 45S rDNA clusters were observed on some of these chromosomes (Figure 3e,f).

Thus, the 45S and 5S rDNA clusters revealed in the karyotypes of the species from the sect. *Hedysarum* could serve as species-specific markers in taxonomic studies. In the sect. *Multicaulia*, variability in a number of minor 45S rDNA loci was detected only in *H. zundukii* [39]. For more precise chromosome identification and also clarification of the *Multicaulia* species relationships, other chromosome molecular markers, such as satellite DNAs, were required.

### 2.4. Molecular and Cytogenetic Characterization of Repeatomes of the Species from the Section Multicaulia

Repetitive DNA sequences (DNAs) are the main components of the genome in vascular plants [68,69,70]. Both dispersed (DNA transposons and retrotransposons) and tandemly arranged (ribosomal DNA and satellite DNAs) DNAs are mainly responsible for genome size variations [71,72,73]. In the diploid *Hedysarum* species with 2n = 2x = 16, the amount of nuclear DNA ranged within 2C = 1.26–3.4 pg [60], which was relatively small for plants [74,75,76]. In various plant taxa, including Fabaceae species, tandem DNA repeats were used as chromosomal markers to study intra- and interspecific genome diversity, reveal chromosomal rearrangements, and also to clarify their evolutionary pathways [77,78,79,80].

**Figure 4:**
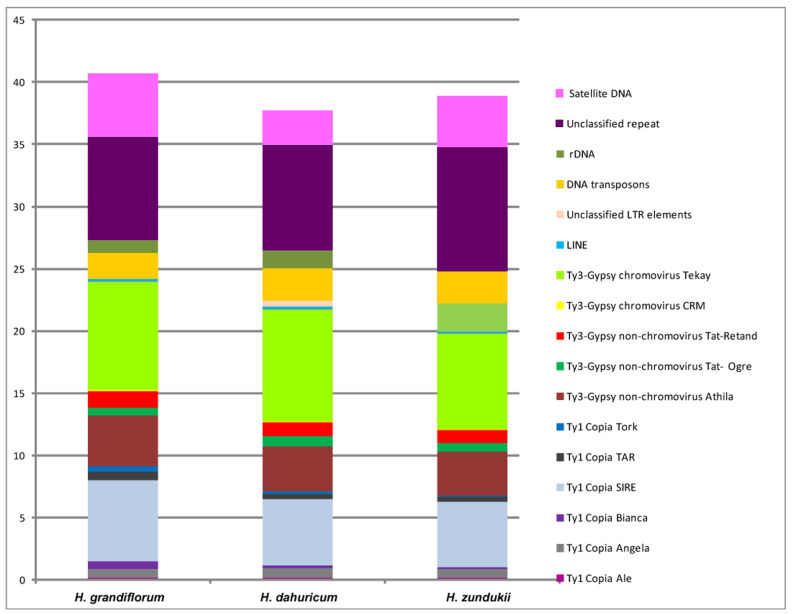
Genome proportion of most abundant DNA repeats in *H. grandiflorum*, *H. dahuricum*, and *H. zundukii*. The genome proportion of individual repeat types was obtained as a ratio of reads specific to individual repeat types to all reads used for clustering analyses by the RepeatExplorer pipelines. The figure is adapted from “Integration of Genomic and Cytogenetic Data on Tandem DNAs for Analyzing the Genome Diversity Within the Genus *Hedysarum* L. (Fabaceae)” by Yurkevich et al., 2022, *Frontiers in Plant Science*, *13*, 865958 [39].

Comparative studies of repeatomes of related species provide new insight into the organization and divergence of their genomes, and, currently, intra- and interspecific variability in repeated DNA content is being extensively studied [81,82,83]. Recently, the comparative analysis of repeatomes of *H. grandiflorum*, *H. zundukii*, and *H. dahuricum* (sect. *Multicaulia*) was performed, which included DNA sequencing of their genomes based on NGS technology and the genome-wide bioinformatic analysis by RepeatExplorer/TAREAN pipelines [39]. In *H. grandiflorum*, *H. zundukii*, and *H. dahuricum*, a high similarity in the composition of their repeatomes was revealed. According to the results of RepeatExplorer/TAREAN analysis, transposable elements (TEs) made up the majority of their repetitive DNAs (Figure 4). Depending on the species, 20–24% of the revealed TEs belonged to retrotransposons (Class I), and about 2.0–2.5% of the TEs belonged to transposons (Class II).

**Figure 5:**
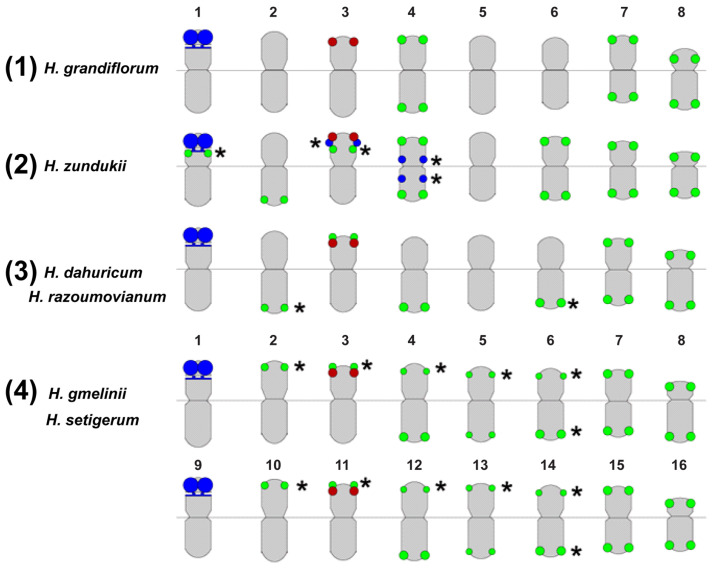
Generalized idiograms of *Hedysarum* chromosomes showing the chromosomal distribution of the examined markers: Hz 6 (green), 45S rDNA (blue), and 5S rDNA (red). Asterisks indicate polymorphic sites. The figure is adapted from “Integration of Genomic and Cytogenetic Data on Tandem DNAs for Analyzing the Genome Diversity Within the Genus *Hedysarum* L. (Fabaceae)” by Yurkevich et al., 2022, *Frontiers in Plant Science*, *13*, 865958 [39].

In the genomes of eukaryotes, retrotransposons (Class I) are the most abundant transposable elements [68,69,70,81]. Within the legume family, their composition can vary in different species due to the predominant number of Ty1 Copia [84,85] or Ty3-Gypsy elements [70,86]. The content of ribosomal DNA represented 1.0–2.6% of the genomes of *H. grandiflorum*, *H. zundukii*, and *H. dahuricum*. Satellite DNA makes up a small proportion of their genome (2.68–5.09%), and the largest amount was found in *H. grandiflorum* [39]. Most Fabaceae species were characterized by a large number of different satDNAs [79,81]. In different satDNAs, a rather high rate of genomic changes was revealed, and, moreover, satDNAs were either species-specific or common to a certain group of related species [81,83]. Using TAREAN, promising putative DNA satellites, which could be potential cytogenetic markers for *Hedysarum*, were identified [39]. Despite the fact that the number of identified tandem DNAs was different in *H. grandiflorum*, *H. zundukii*, and *H. dahuricum,* the main set of common tandem DNA repeats was homologous, and their monomer sequences were mostly identical in length, which confirmed close genomic relationships between these species. Taking this into account, the most abundant satDNAs identified in the repeatome of *H. zundukii* (Hz 2, Hz 6, Hz 9, Hz 44, and Hz 96) were used as potential cytogenetic markers and then mapped by FISH to chromosomes of several species of the sect. *Multicaulia* [39]. Hz 6 presented specific chromosome distribution patterns demonstrating permanent clusters localized in the subtelomeric regions of three chromosome pairs (4, 7, and 8) and several polymorphic clusters observed on the remaining chromosome pairs. Combination of Hz 6 with any of the pericentromeric clusters of Hz 9, Hz 2, Hz 96, or Hz 44 made it possible to identify all homologous chromosomes in karyotypes of *H. grandiflorum*, *H. zundukii*, *H. dahuricum, H. razoumovianum, H. gmelinii,* and *H. setigerum,* and also to analyze intra- and interspecific genome variability within the sect. *Multicaulia* (Figure 5).

The analysis of patterns of chromosome distribution of the examined molecular markers (45S rDNA, 5S rDNA, and the satDNAs) showed that the studied species could be subdivided into four karyological groups: (1) *H. grandiflorum* (subsection *Subacaulia*), (2) *H. zundukii* (subsection *Subacaulia*), (3) *H. razoumovianum*, *H. dahuricum* (subsection *Multicaulia*), *H. gmelinii,* and *H. setigerum* (subsection *Multicaulia*) (4) (Figure 5) [39]. The similarity of chromosome distribution patterns of the satDNAs observed in *H. setigerum* and *H. gmelinii*, confirmed the earlier reported taxonomic status of *H. setigerum* as a subspecies of *H. gmelinii* [16,19]. Thus, the comprehensive molecular cytogenetic analysis of six species from the sect. *Multicaulia* revealed a close relationship among their genomes (regardless of the regions of their growth and the range sizes), indicating that they have a common origin.

### 2.5. Comparative Analysis of Genomes of Hedysarum Species by RapidGISH

**Figure 6:**
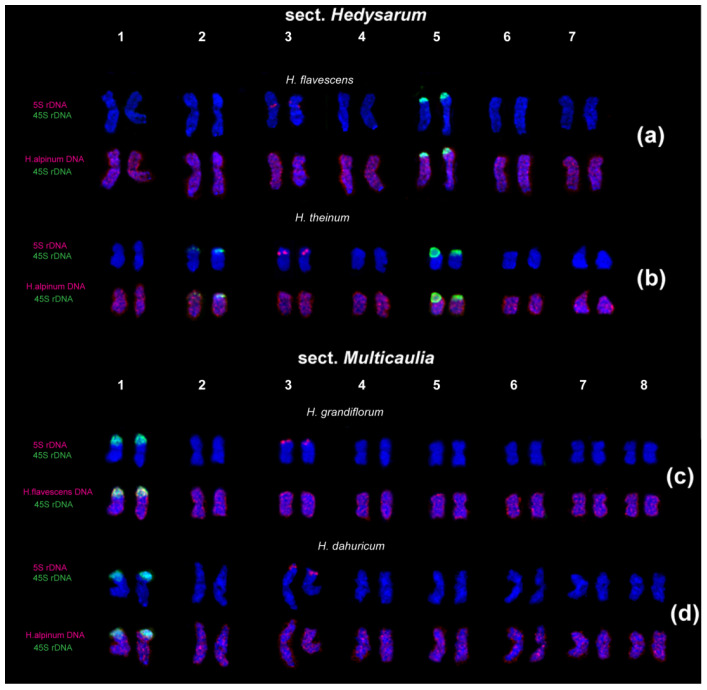
Karyograms of the *H. flavescens* (**a**), *H. theinum* (**b**), *H. grandiflorum* (**c**), and *H. dahuricum* (**d**) after FISH with 45S rDNA (green) and 5S rDNA (red), and also rapidGISH with genomic DNA of *H. flavescens* and/or *H. alpinum* (red). Chromosome DAPI-staining—blue. The figure is adapted from “Comparative analysis of genomes of six species of *Hedysarum* L. (Fabaceae) by the rapidGISH technique” by Yurkevich et al., 2023, *Problems of Botany in Southern Siberia and Mongolia 22*, 436–440 [92].

Comparison of the species genomes using genomic hybridization in situ (GISH) makes it possible to identify homologous DNA sequences on chromosomes of the related species. Therefore, GISH is an important approach to understanding the processes of speciation and clarifying the phylogenetic relationship between plant taxonomic groups [87,88,89,90]. The variant of rapidGISH reveals common tandem repeats and clearly demonstrates the patterns of their distribution on plant chromosomes [91]. A comparative analysis of genomes of several species from the sections *Hedysarum* and *Multicaulia* was carried out by rapidGISH. As labeled probes in the rapid GISH assays, genomic DNAs of *H. flavescens* and *H. alpinum* were used (Figure 6) [92], since *H. flavescens* is considered to be the closest species to one of the hypothetical ancestral species [10], and *H. alpinum* is the most common species of the sect. *Hedysarum* in Eurasia [1,16,18]. A dispersed-clustered distribution of hybridization signals was revealed on the chromosomes of the species from the sect. *Hedysarum* (Figure 6a,b). At the same time, only weak dispersed signals were observed on chromosomes of the species belonging to the sect. *Multicaulia* (Figure 6c,d). These data indicated the presence of a small number of homologous DNA sequences in the genomes of the species from the sections *Hedysarum* and *Multicaulia*. Additionally, the rapidGISH analysis demonstrated a closer relationship among genomes of the species from the same section than those belonging to different sections.

### 2.6. Phylogeny of the Genus Hedysarum

The phylogenetic studies performed with the use of nuclear (ITS) and plastid DNA sequences have significantly accelerated the investigation of the evolutionary path of the genus *Hedysarum*. Particularly, a new revision of this genus based on molecular data (nrDNA ITS, plastid trnL-F, and matK) was conducted by Amirahmadi et al. [30]. After the phylogenetic reconstructions, they separated *H.* sect. *Membranacea* from *Hedysarum* and established a new genus, *Greuteria* Amirahmadi & Kaz. Osaloo, and also transferred *Sartoria* Boiss. & Heldr. into *Hedysarum* [30].

Phylogenetic analyses based on nuclear and plastid DNA sequences identified the genus *Hedysarum* with three well-supported clades redefined as sections *Hedysarum*, *Stracheya*, and *Multicaulia* [13,14,15,35]. According to the phylogenetic studies conducted with the use of nuclear (ITS, ETS, PGDH, TRPT, SQD1) and plastid (trnH–psbA, trnC–petN, trnL–trnF, trnS–trnG, petN–psbM) DNA sequences, two main lineages in the genus *Hedysarum*, the *Hedysarum* s.s. clade (*H*. sect. *Hedysarum* and *H.* sect. *Stracheya*) and the *Sartoria* clade (*H.* sect. *Multicaulia*), are presented [14]. At the same time, Duan et al. [13] and Nafisi et al. [15] placed the *H*. sect. *Stracheya* together with the *H*. sect. *Multicaulia* based on the analysis of nuclear markers. On the other side, the phylogenetic studies of Central Asian *Hedysarum* species conducted using a combined plastid dataset (matK, trnL-F, psbA-trnH) [35] revealed the *H*. sect. *Stracheya* as sister to the *H*. sect. *Hedysarum* and the *H*. sect. *Multicaulia*, which was different from all previous phylogenetic reports [13,14,15]. Therefore, the close relationship between *H*. sect. *Stracheya*, *H*. sect. *Hedysarum*, and *H*. sect. *Multicaulia* needs to be further investigated using the whole plastid genome.

Duan et al. [13], using nrDNA ITS and three plastid regions (matK, trnL–F, trnH–psbA), recognized three clades within the genus *Hedysarum*. However, the relationships within the sect. *Multicaulia* were not well resolved [13,14]. The *H*. sect. *Multicaulia* was taxonomically subdivided into three subsections, *Multicaulia*, *Subacaulia,* and *Crinifera* [2]. At the same time, based on nuclear (ITS) and plastid DNA sequences (trnL–trnF and matK), two distinct lineages, the subsects. *Multicaulia* and *Crinifera*, were revealed in the *H*. sect. *Multicaulia*, and the subsect. *Subacaulia* was not recognized [15,35]. Additionally, many species within the subsect. *Crinifera* remained unresolved, and several subgroups of species were formed [14,35].

Phylogenetic analyses of the genus *Hedysarum* performed based on both nuclear and plastid datasets showed that the genus was paraphyletic [30]. Moreover, Duan et al. [13] and Liu et al. [14] demonstrated that the genus *Hedysarum* was paraphyletic according to the nuclear tree but monophyletic based on the plastid data. This discrepancy between the nuclear and plastid trees could be explained by the hypothesis of chloroplast capture via introgression [13,14,30].

At the same time, each of the three sections, *Hedysarum*, *Stracheya,* and *Multicaulia*, was congruently identified as monophyletic [13,14]. Additionally, the results of phylogenetic studies did not support the monophyly of each of the three subsections of the sect. *Multicaulia*: the sect. *Multicaulia* was subdivided into two distinct lineages, which were taxonomically redefined as *H*. subsects. *Multicaulia* and *Crinifera* [15].

The molecular phylogenetic data demonstrating the paraphyly of *Hedysarum* might explain the differences in basic chromosome numbers revealed among the sections of this genus [13,14,30]. Moreover, the species are taxonomically grouped according to their basic chromosome numbers x = 7 (*H*. sect. *Hedysarum*) or x = 8 (*H*. sect. *Multicaulia* and *H*. sect. *Stracheya*) [2,28]. The results of rapid GISH assays demonstrating the presence of homologous DNA sequences in species genomes from both *Hedysarum* and *Multicaulia* sections might be due to the existence of their common ancestor and do not exclude paraphyletic origin of the genus [14,30,35,92].

According to the latest analyses of nrDNA ITS and plastid datasets, West Asia is the most probable region of the origin of the species from the sect. *Multicaulia*. East Asia appears to be the center of the origin of the species from the sect. *Hedysarum* [35]. Biogeographic analyses indicate that *Hedysarum* species most likely originated in West Asia and/or East Asia during the Early Miocene or Middle Miocene and then distributed to adjacent areas of Eurasia as well as North America via the Bering Land Bridge [15,35].

## 3. Conclusions

The genus *Hedysarum*, which includes economically valuable species, is one of the most systematically complicated groups in the legume family (Fabaceae). The use of modern approaches, including molecular phylogenetic analyses and examination of the species repeatomes and their chromosome organization, made it possible to specify the taxonomy and origin of this genus, as well as clarify the genome relationships between the species and within the sections *Hedysarum* and *Multicaulia*.

## Data Availability

All data generated or analyzed during this study are contained within the article.
